# Impaired fasting glucose: a risk factor for atrial fibrillation and heart failure

**DOI:** 10.1186/s12933-021-01422-3

**Published:** 2021-11-24

**Authors:** Viktor Lind, Niklas Hammar, Pia Lundman, Leif Friberg, Mats Talbäck, Göran Walldius, Anna Norhammar

**Affiliations:** 1 Division of Cardiovascular Medicine, Department of Clinical Sciences, Danderyd Hospital, Karolinska Institutet, 182 88 Stockholm, Sweden; 2 Department of Cardiology, Danderyd University Hospital, Stockholm, Sweden; 3 Unit of Epidemiology, Institute of Environmental Medicine, Karolinska Institutet, Stockholm, Sweden; 4 Cardiology Unit, Department of Medicine K2, Karolinska Institutet, Stockholm, Sweden; 5 Capio S:T Görans Hospital, Stockholm, Sweden

**Keywords:** Prediabetes, Impaired fasting glucose, Dysglycaemia, Diabetes, Cardiovascular disease, Atrial fibrillation, Heart failure

## Abstract

**Background:**

Dysglycaemia is associated with overall cardiovascular disease even at prediabetes levels. The aim of this study was to explore the association between glucose levels and future risk of developing atrial fibrillation and heart failure, respectively.

**Methods:**

In this prospective cohort study subjects from the Swedish AMORIS-cohort with fasting glucose from health examinations 1985–1996 without previous cardiovascular disease (N = 294,057) were followed to 31 December 2011 for incident atrial fibrillation or heart failure. Cox proportional hazard models with attained age as timescale and adjustments for sex, cholesterol, triglycerides, and socioeconomic status were used to estimate hazard ratios by glucose categorized groups (normal glucose 3.9–6.0 mmol/L, impaired fasting glucose; 6.1–6.9 mmol/L, undiagnosed diabetes ≥ 7.0 mmol/L, and diagnosed diabetes).

**Results:**

During a mean follow-up time of 19.1 years 28,233 individuals developed atrial fibrillation and 25,604 developed heart failure. The HR for atrial fibrillation was 1.19 (95% confidence interval 1.13–1.26) for impaired fasting glucose, 1.23 (1.15–1.32) for undiagnosed diabetes and 1.30 (1.21–1.41) for diagnosed diabetes. Corresponding figures for heart failure were; 1.40 (1.33–1.48), 2.11 (1.99–2.23), 2.22 (2.08–2.36) respectively. In a subset with BMI data (19%), these associations were attenuated and for atrial fibrillation only remained statistically significant among subjects with diagnosed diabetes (HR 1.25; 1.02–1.53).

**Conclusions:**

Fasting glucose at prediabetes levels is associated with development of atrial fibrillation and heart failure. To some extent increased BMI may drive this association.

**Supplementary Information:**

The online version contains supplementary material available at 10.1186/s12933-021-01422-3.

## Background

Diabetes mellitus is associated with an increased risk of cardiovascular disease and compromised longevity [1]. This increased risk is present already at prediabetes levels [2]. Globally, diabetes is estimated to affect 463 million adults, with one-third to one half being undiagnosed, and with a projected increase in prevalence to 578 million in the year 2030 [3]. One of the most severe cardiovascular complications associated with diabetes is heart failure [4], with diabetes increasing the risk for heart failure by up to two times when risk factors are well-controlled and substantially higher if uncontrolled [1, 5, 6]. Furthermore, diabetes has been implicated as a risk factor for incident atrial fibrillation, as well as having a negative impact on prognosis of this disease [6–10]. The cardiovascular risk associated with prediabetes is mostly described for cardiovascular mortality, but an association has also been suggested for heart failure [11, 12] and atrial fibrillation [13–16].

The aim of this study was to explore the association between glucose levels, including those below the current diagnostic threshold of diabetes, and future risk of developing atrial fibrillation and heart failure, respectively, among individuals without previous cardiovascular disease.

## Methods

### Study population and data sources

**Fig. 1:**
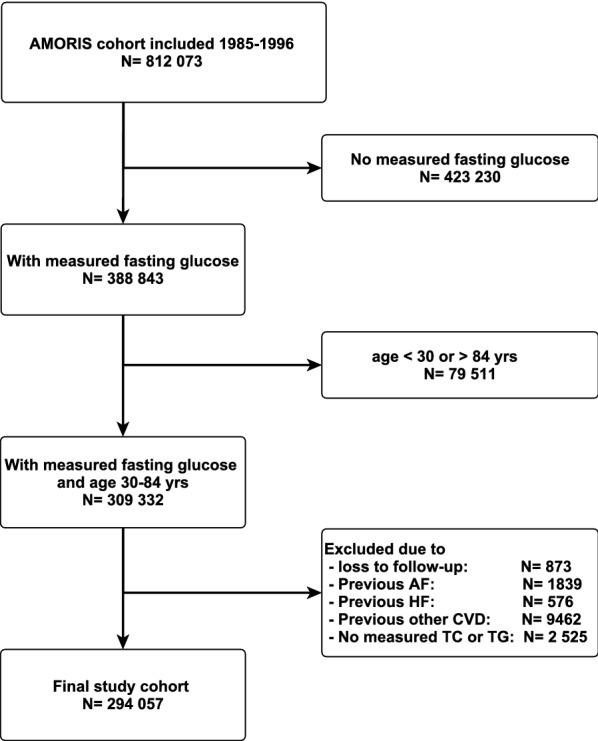
Flowchart presenting exclusion from original AMORIS cohort to the final study cohort. *AF* Atrial fibrillation, *HF* Heart failure, *CVD* Cardiovascular disease, *TC* Total cholesterol, *TG* Triglycerides

This prospective cohort study included subjects from the Swedish Apolipoprotein MOrtality RISk (AMORIS) cohort [17]. The cohort was initially set up to study associations between apolipoprotein (apo) B and apoA-1 and cardiovascular disease, but has since then been extensively utilized to study metabolic disturbances and inflammation and the risk of several chronic diseases including type 2 diabetes [18]. It consists of 812 073 unselected individuals predominantly from the larger Stockholm area referred for laboratory testing either via routine health check-ups from occupational health care or from primary health care during the time-period 1985 to 1996. From this cohort we included study subjects 30–84 years of age with a measurement of fasting glucose, triglycerides, and total cholesterol at the same examination (index examination). Subjects with a history of atrial fibrillation, heart failure, ischemic heart disease, stroke or revascularization at the index examination were excluded (See Additional file 1: Table S1). The final study population included 294 057 subjects (Fig. 1). Information on co-morbidities and mortality prior to and following the index examination was gained through linkage to the National Patient register going back to 1964 regionally, to 1972 in Stockholm county, and to 1987 nationally, the National Cancer Register going back to 1958 and the National Cause of Death Register going back to 1961. For record linkage we utilized the unique Swedish personal identification number available in all relevant registers.

### Laboratory measurements

Information on biomarkers reflecting metabolic abnormalities and inflammation was obtained from the index examination. All biomarkers were analyzed at the time of the health examination in fresh blood at the CALAB medical laboratory (Stockholm, Sweden). For details on analytic methods, see Additional File 1.

### Follow-up

Subjects were followed for incident atrial fibrillation and incident heart failure respectively using the Patient Register and the Cause of Death Register. ICD-codes were registered by physicians from inpatient care, emergency care and, from 2001, from specialized outpatient care (See Additional File 1: Table S2). Follow-up started at the index examination and ended at the first recorded diagnosis of atrial fibrillation or heart failure respectively, death, migration or end of follow-up at December 31, 2011.

### Definitions

*Outcomes:* First hospitalization or specialized outpatient visit or main cause of death due to atrial fibrillation or heart failure respectively. *Combined event* was defined as first event of either atrial fibrillation or heart failure.

*Exposure:* Subjects free of diagnosed diabetes at baseline were categorized into four groups by fasting glucose measurements at the index examination; (i) *Low* (< 3.9 mmol/L); (ii) *Normal* (3.9–6.0 mmol/L); (iii) *Impaired Fasting Glucose* (6.1–6.9 mmol/L in accordance to the definition by the WHO)); [19] (iv) *Undiagnosed diabetes* (≥ 7.0 mmol/L).

*Diagnosed prevalent diabetes* was defined as a registered ICD-code of diabetes prior to the index examination in the National Diabetes Register or the Patient Register (250) or as self-reported diabetes in linked research studies [17].

*Prediabetes* was defined as a fasting glucose between 6.1 and 6.9 mmol/L, but not on 2 h oral glucose tolerance test or HbA1c since these were not available.

*eGFR (estimated glomerular filtration rate)* was calculated using the Chronic Kidney Disease Epidemiology Collaboration formula (CKD-EPI) [20].

*BMI (Body Mass Index)* calculated as kg/m^2^ was attained from either the index examination, earlier examinations, the National Medical Birth Register, the Diabetes Register or linked research studies. We used BMI within a time window of up to ten years prior to the index examination.

*Co-morbidities:* Co-morbidities were identified from ICD-codes in the Patient Register or from diagnosed malignant cancer in the Cancer Register (See Additional File 1: Table S3).

*Socioeconomic status*: Information on socioeconomic status was obtained from the nearest national population and housing census 1970–1990 prior to the index examination. Socioeconomic status was classified as white- or blue-collar worker based on a Swedish Socioeconomic Index (SEI).

### Statistical analysis

Baseline characteristics were described using frequencies and percentages for categorical variables and interquartile range and median values for continuous variables. Incidences were calculated as the numbers of new events divided by person years at risk. Cox proportional hazards regression with attained age as timescale was used to estimate hazard ratios (HR) together with 95% confidence intervals (CI). Adjustments were made for sex and with additional adjustment for total cholesterol, triglycerides and socioeconomic status. Corresponding sensitivity analysis was performed with additional adjustment for BMI in a sub-population. To illustrate the possible impact of competing events, the incidence proportion of the first event of atrial fibrillation, heart failure and death due to other causes, respectively, were calculated by age groups across a 25-year follow-up period and shown graphically. Proportional hazard ratios for atrial fibrillation and heart failure respectively, in relation to increasing glucose levels were estimated using restricted cubic splines together with 95% confidence intervals (mean glucose equal 5 mmol/L served as the reference with HR of 1.0).

Statistical analyses were conducted using STATA version 14.2 (StataCorp LLC, College Station, TX, USA).

## Results

The cohort had a median age of 47 years, 46% were women and 92% were born in a Nordic country. The mean follow-up time was 19.1 years. The overall median fasting glucose was 4.8 mmol/L (IQR 4.5–5.2 mmol/L). Impaired fasting glucose and diabetes was associated with higher age and male sex (Table 1). Triglyceride-levels were increased among subjects with impaired fasting glucose, diagnosed diabetes and with undiagnosed diabetes compared to those with normal glucose levels. The apo-B/apo-A1 ratio was also higher in all groups with elevated glucose levels. The overall proportion of subjects with an eGFR below 60 ml/min/1.73 m^2^ was 3.5%, among those with normal glucose levels 3.2%, and among subjects with undiagnosed diabetes 10.3%. Information on BMI was available in only 19% of subjects. This subgroup had similar background characteristics as the total cohort (See Additional File 1: Table S4). The highest mean BMI was found in the group with previously undiagnosed diabetes.

**Table 1:** Baseline characteristics

	N	Total N = 294 057	Low < 3.9 mmol/L n = 7 408 (2.5%)	Normal 3.9–6.0 mmol/L n = 266 943 (90.8%)	IFG 6.1–6.9 mmol/L n = 9 682 (3.3%)	Undiagnosed DM ≥ 7.0 mmol/L n = 5 457 (1.9%)	**Diagnosed DM** n = 4 567 (1.6%)
Age (years)	294 057	47 (40–56)	43 (36–51)	47 (39–55)	54 (46–61)	57 (49–64)	53 (45–60)
Sex, female	294 057	135 562 (46.1)	4 382 (59.2)	124 497 (46.6)	3 300 (34.1)	1 646 (30.2)	1 737 (38.0)
Blue collar worker	286 058	157 549 (55.1)	4 090 (56.8)	142 258 (54.7)	5 464 (58.3)	3 084 (59.6)	2 653 (60.1)
Born in Nordic countries	294 057	270 242 (92.1)	6854 (92.5)	245 489 (92.0)	8 878 (91.7)	4 884 (89.5)	4 147 (90.8)
Referred from occupational health care	259 549	193 573 (74.6)	5 187 (79.0)	177 643 (75.4)	5 585 (65.6)	2 710 (57.2)	2 448 (59.9)
BMI (kg/m2)	55 390	24.0 (21.8–26.5)	22.4 (20.6–24.6)	24.0 (21.8–26.3)	26.5 (24.2–29.5)	28.0 (25.5–30.9)	26.6 (23.4–29.8)
Kidney disease	294 057	288 (0.1)	9 (0.1)	257 (0.1)	9 (0.1)	2 (0.0)	11 (0.2)
Liver disease	294 057	1 102 (0.4)	38 (0.5)	862 (0.3)	65 (0.7)	51 (0.9)	86 (1.9)
Asthma/COPD	294 057	2 026 (0.7)	63 (0.9)	1 740 (0.7)	93 (1.0)	55 (1.0)	75 (1.6)
History of cancer	294 057	6 550 (2.2)	131 (1.8)	5 812 (2.2)	287 (3.0)	196 (3.6)	124 (2.7)
Mitral stenosis	294 057	34 (0)	0	30 (0)	1 (0)	2 (0)	1 (0)
Mechanical valve-replacement	294 057	25 (0)	0	24 (0)	1 (0)	0	0
Fasting glucose, mmol/L	294 057	4.8 (4.5–5.2)	3.7 (3.5–3.8)	4.8 (4.5–5.2)	6.3 (6.2–6.6)	8.2 (7.3–10.4)	8.3 (6.2–11.9)
Fructosamine, mmol/L	242 205	2.1 (2.0–2.2)	2.0 (1.9–2.2)	2.1 (2.0–2.2)	2.2 (2.0–2.3)	2.5 (2.2–2.9)	2.6 (2.3–3.1)
Total cholesterol, mmol/L	294 057	5.7 (5.0–6.4)	5.4 (4.7–6.1)	5.7 (5.0–6.4)	6.0 (5.3–6.8)	6.0 (5.3–6.8)	5.8 (5.0–6.6)
Triglyceride level, mmol/L	294 057	1.0 (0.7–1.5)	0.9 (0.6–1.2)	1.0 (0.7–1.5)	1.5 (1.0–2.2)	1.9 (1.3–2.8)	1.5 (1.0–2.4)
Apo-B/Apo-A1 ratio	294 057	0.89 (0.71–1.10)	0.82 (0.67–1.01)	0.88 (0.71–1.09)	0.96 (0.79–1.21)	1.05 (0.85–1.28)	0.97 (0.78–1.22)
Haemoglobin, g/L	73 119	141.0 (133.0–150.0)	138.0 (129.0–147.0)	141.0 (132.0–150.0)	146.0 (138.0–154.0)	149.0 (140.0- 157.0)	145.0 (136.0–154.0)
WBC, 10^9/L	67 806	6.1 (5.1–7.4)	6.1 (5.1–7.6)	6.1 (5.1–7.3)	6.6 (5.6–7.9)	6.9 (5.8–8.3)	6.6 (5.5–8.0)
Subjects with eGFR below 60 ml/min/1.73 m^2^	283 448	10 173 (3.5)	180 (2.4)	8 435 (3.2)	698 (7.2)	559 (10.3)	301 (6.6)
Uric acid, umol/L	278 499	287.0 (240.0–337.0)	262.0 (218.0–311.0)	286.0 (239.0–335.0)	335.0 (285.0–389.0)	321.0 (271.0–380.0)	286.0 (235.0–345.0)
CRP, mg/L	139 257	4.0 (1.0–6.0)	3.0 (1.0–6.0)	4.0 (1.0–6.0)	4.0 (3.0–7.0)	4.0 (2.0–8.0)	4.0 (2.0–7.0)
Haptoglobin, g/L	234 448	1.0 (0.9–1.2)	1.0 (0.8–1.2)	1.0 (0.9–1.2)	1.1 (1.0–1.4)	1.2 (1.0–1.4)	1.1 (0.9–1.3)

Categorical variables presented as absolute and relative (percentages) frequencies and continuous variables as median and interquartile range

*IFG* Impaired fasting glucose, *DM* Diabetes mellitus, *COPD* Chronic obstructive pulmonary disease, *WBC* White blood cells

### Outcome

**Fig. 2:**
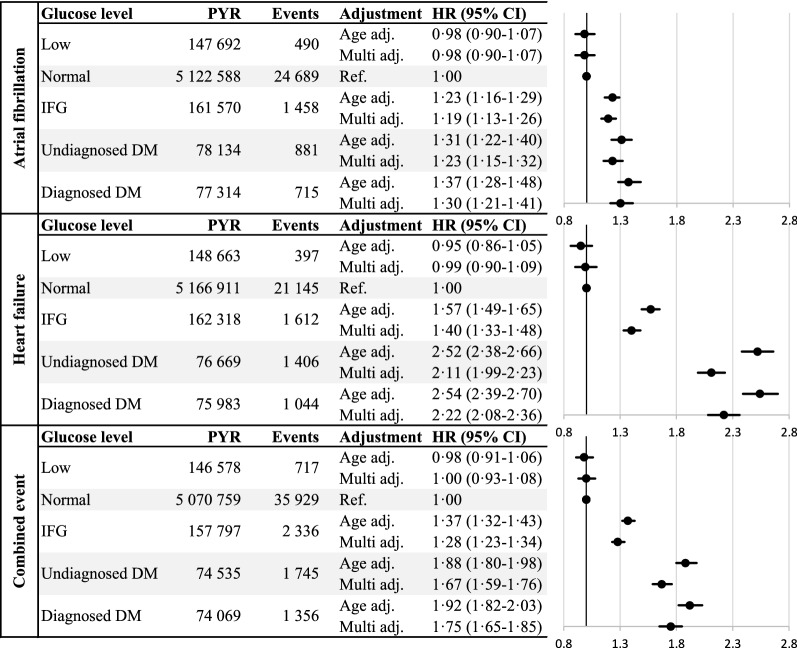
Total events, and hazard ratios (HR) for atrial fibrillation, heart failure and combined event by glucose group. Hazard ratios are presented as adjusted for attained age as timescale and sex (Age adj.) and further adjusted for total cholesterol, total triglyceride levels and socioeconomic index (Multi adj.). *PYR* Person-years under risk, *IFG* Impaired fasting glucose, *DM* Diabetes mellitus

During the follow-up period there were 28 233 new cases of atrial fibrillation, 25 604 new cases of heart failure and 42 083 combined events observed. Elevated fasting glucose was associated with an increased risk for atrial fibrillation, heart failure, and a combined event respectively. The incidence of atrial fibrillation was increased among all groups with elevated glucose measurements (Fig. 2). The highest incidence was found in the group with already diagnosed diabetes followed by the groups undiagnosed diabetes and impaired fasting glucose. The incidence of heart failure followed the same pattern although with higher hazard ratio estimates.

Further adjustment for BMI (Table 2) attenuated the associations for both outcomes and in particular for atrial fibrillation, where the association only remained statistically significant among subjects with diagnosed diabetes (HR 1.25; 1.02–1.53).

**Table 2:** Sensitivity analysis in a subgroup of 55 390 subjects with BMI available, stratified by fasting glucose levels

Glucose level	Atrial fibrillation 4 167 events		Heart failure 3 138 events	
	Events	HR [95% CI]	Events	HR [95% CI]
Low n = 1622 (2.9%)	75	0.88 [0.70–1.10]	51	0.88 [0.67–1.16]
Normal n = 50,793 (91.7%)	3 713	1.00	2 628	1.00
IFG n = 1537 (2.8%)	187	1.12 [0.96–1.30]	175	1.34 [1.15–1.57]
Undiagnosed DM n = 705 (1.3%)	95	1.25 [1.02–1.54]	158	2.82 [2.39–3.32]
Diagnosed DM n = 733 (1.3%)	97	1.36 [1.11–1.67]	126	2.54 [2.11–3.04]
After further adjustment for BMI				
Low n = 1 622 (2.9%)	75	0.93 [0.74–1.17]	51	0.93 [0.70–1.22]
Normal n = 50,793 (91.7%)	3 713	1.00	2 628	1.00
IFG n = 1537 (2.8%)	187	1.03 [0.89–1.19]	175	1.25 [1.07–1.46]
Undiagnosed DM n = 705 (1.3%)	95	1.11 [0.90–1.36]	158	2.51 [2.12–2.96]
Diagnosed DM n = 733 (1.3%)	97	1.25 [1.02–1.53]	126	2.36 [1.96–2.83]

Hazard ratios and 95% confidence limits for the events adjusted for

attained age as timescale, sex, total cholesterol, total triglyceride levels and socioeconomic index shown on top with further adjustment for BMI below

*IFG* Impaired fasting glucose, *DM* Diabetes mellitus

**Fig. 3:**
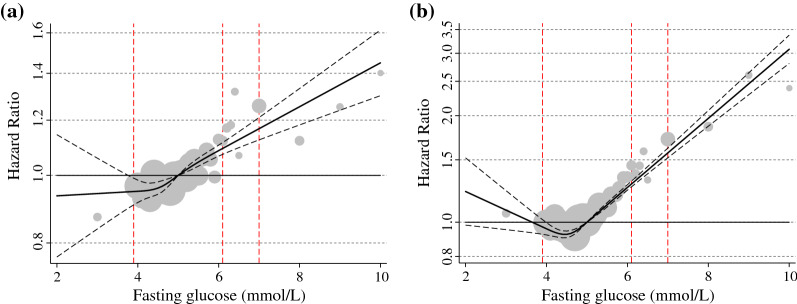
Hazard ratio and 95% confidence limits for **A** atrial fibrillation (left) and **B** heart failure (right) by fasting glucose level presented as splines. Subjects with a fasting glucose above or below 10 standard deviations not presented. Hazard ratios are adjusted for attained age as timescale, sex, total cholesterol, total triglyceride levels and socioeconomic index. The dotted lines indicate the 95% confidence interval. The grey spheres reflect the relative size of the number of subjects with respective level of fasting glucose and the red vertical lines show the cut-off value for low glucose (3.9 mmol/L), and the lower and higher limit for impaired fasting glucose (6.1 mmol/L and 6.9 mmol/L) respectively

The spline curves (HR and 95% CI) for atrial fibrillation and heart failure showed a continuous increased risk of both incident atrial fibrillation and incident heart failure with successively increasing glucose levels (Fig. 3). At a fasting glucose of 6.1 mmol/L the HR (95% CI) for atrial fibrillation was 1.09 (1.07–1.11).

**Fig. 4:**
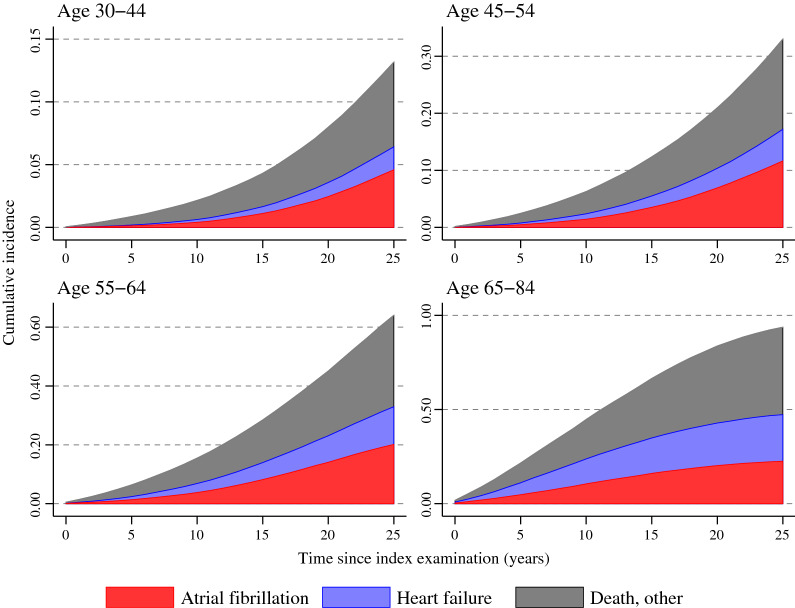
Cumulative incidence of events. Cumulative incidence (shown as surface area of each curve) for men and women of first event of atrial fibrillation (red), heart failure (blue) or death due to other causes (black) during 25 years of follow-up in different age groups. Observe the different scales on the y-axis between the age groups

Figure 4 illustrates the incidence proportions of atrial fibrillation, heart failure and death for men and women by age groups and follow-up time. Among those below the age of 65 years at inclusion atrial fibrillation occurred more frequently as first event without previous heart failure, whereas among those aged 65 years and older atrial fibrillation and heart failure occurred as first event in approximately equal proportions. In the oldest age group mortality from other causes was common and at the end of the follow-up most subjects had experienced at least one of these outcomes.

## Discussion

In this long-term follow-up of atrial fibrillation and heart failure in subjects free from cardiovascular disease at baseline there are three major findings. First, that increasing glucose values are associated with an increased risk of future development of both atrial fibrillation and heart failure, even at levels below the diagnostic diabetes threshold. Second, the association between glucose and heart failure is stronger than the association to atrial fibrillation. Third, the association between glucose and atrial fibrillation could at least in part be due to or mediated by increased BMI, i.e. overweight and obesity, main risk factors of impaired fasting glucose and type 2 diabetes.

The main strength of this study is the large size of this population-based cohort without previous cardiovascular disease. The cohort was followed for a mean of nearly two decades, with high quality national registries ensuring essentially no loss of follow-up. Furthermore, all blood samples were analyzed on fresh blood in one single laboratory using consistently implemented and well documented methods, ensuring high quality and comparable measurements.

However, information on BMI was limited to a sub-population and information on waist circumference was lacking, thus the sub-analyses adjusting for BMI must be interpreted with caution. Information on hypertension, current medication, blood pressure and other life-style factors was limited and could not be included. As these are all potential confounding factors, and hypertension an established risk factor for both atrial fibrillation and heart failure, this is an important limitation. Levels of HbA1c was only available for a small proportion of the cohort, as this was not routinely analyzed at the time of inclusion. Information on type of diabetes was not available. The majority of individuals with diabetes in Sweden at time of inclusion had type 2 diabetes, but since the risk for cardiovascular disease is higher in type 1 diabetes this could have caused a minor overestimation of risk. For identification of subjects with diabetes two national registries were used to achieve a high level of completeness. However, it is possible that some subjects with diabetes lacked a registered diagnosis. Since we did not have information regarding medication, it is possible that some subjects with well controlled diabetes and lack of registered diagnoses were misclassified as not having diabetes, which may have slightly diluted actual hazard ratios. Furthermore, information on type of heart failure was not available, but all cases of heart failure required either hospitalization or specialized outpatient care. As the majority (92%) of subjects were born in a Nordic country it is likely that the ethnicity was mainly Caucasian, limiting the generalizability of this study.

It is well known from several epidemiological cohort studies that glucose levels even below the diagnostic diabetes threshold are associated with coronary heart disease, cardiovascular mortality and total mortality [21]. The present study shows that there also is a graded association between glucose and atrial fibrillation and heart failure, starting already at prediabetes levels. This is also supported in a recent meta-analysis on cohort studies in diabetes and prediabetes [16]. However, we believe that our finding of a continuous association of glucose levels and future risk for atrial fibrillation in persons free from previous cardiovascular disease is unique with regards to the large cohort-size and extensive follow-up time and in the same cohort showing associated risks for heart failure. Importantly, atrial fibrillation was present not only in conjunction with or following heart failure, but in the majority of subjects appeared before or in the absence of heart failure. At ages from 65 years and above there was an equal distribution between heart failure and atrial fibrillation as the first occurring event, but in younger subjects atrial fibrillation more frequently appeared as the first of these two outcomes.

Subjects with elevated glucose had higher total cholesterol, triglycerides and apoB-apoA-1 ratios. Information on BMI was available in a subpopulation, among which BMI was higher in all groups with elevated glucose levels. As the association between elevated glucose and in particular atrial fibrillation was attenuated when further adjustment for BMI was made, this could indicate that part of the associated risk might be explained or mediated by obesity and/or presence of the metabolic syndrome. However, as information on BMI was only available in 19% of the cohort and was not consistently recorded at the same date as the glucose level, these results must be interpreted with caution.

The association of diabetes, the metabolic syndrome and obesity to atrial fibrillation has recently gained increased interest [7, 22] with latest ESC guidelines for the management of atrial fibrillation emphasizing lifestyle interventions, including weight control [23]. There are several possible mechanistic explanations for such a relationship. These include atrial remodeling with increased fibrosis [24] and enlarged atrial volume associated with obesity [25]. Factors associated with the metabolic syndrome, such as hypertension, chronic inflammation, increased circulating lipids/free fatty acids, hyperinsulinemia and unhealthy adipose tissue distribution could be contributing pathophysiological mechanisms apart from direct glucose effects [7, 26]. Further, increased pericardial fat accumulation might disturb atrial function [25, 27]. Development of diastolic dysfunction and heart failure with preserved ejection fraction, elevated filling pressures and volume overload of the left atrium could be other potential drivers for the association between dysglycaemic conditions with and without co-existing obesity and atrial fibrillation [22, 28].

The association between elevated glucose levels and heart failure seemed to be stronger than the association to atrial fibrillation, regardless of glucose group. A speculative explanation could be that diabetes affects a majority of important functions and structures involved in the development of heart failure more than atrial fibrillation, as seen in diabetic cardiomyopathy (including myocardial dysmetabolism, coronary heart disease, myocardial fibrosis and hypertrophy) [28].

Of special interest in relation to the association of glucose and atrial fibrillation, is the recently highlighted possibility to prevent development of atrial fibrillation by modifying risk factors associated with dysglycaemia, the metabolic syndrome and a sedentary lifestyle. The ARREST-AF study showed that multifactorial life-style changes in patients with atrial fibrillation resulted in reduced recurrence of atrial fibrillation [29]. In the LEGACY study, weight loss reduced the burden of recurrent atrial fibrillation with contemporary improvement in echocardiographic abnormalities [30]. However, few, if any, have studied primary prevention of atrial fibrillation by modifying dysglycaemia and/or the metabolic syndrome. Interestingly, treatment with SGLT-2 inhibitors among patients with type 2 diabetes seems to decrease development of atrial fibrillation in the DECLARE-TIMI 58 study [31]. A recent retrospective study of patients newly diagnosed with type 2 diabetes and subsequently prescribed SGLT-2 inhibitors showed that the risk for incident atrial fibrillation was significantly lowered, but only among those who achieved a weight loss of of more than 5% [32], implicating the potential to reduce the future prevalence of atrial fibrillation through preventive treatment.

Considering that dysglycaemia is a global epidemic, and that the incidence of atrial fibrillation is increasing worldwide [33], our results emphasize the need for preventive action. There is a need for future studies exploring whether improved glycemic control in individuals with prediabetes affect the risk of future cardiovascular disease.

## Conclusions

Elevated fasting glucose and unrecognized diabetes are risk factors for future development of both atrial fibrillation and heart failure, strengthening that early detection of dysglycaemia is an important risk factor to consider in cardiovascular prevention.

## Supplementary Information

**Additional file 1: Table S1**. International Classification of Diseases [ICD] code 8/9/10 diagnoses and Classification of Surgical Procedures NOMESCO (Nordic Medico-Statistical Committee) codes used to define previous cardiovascular disease as exclusion criteria. **Table S2**. International Classification of Diseases [ICD] code 8/9/10 for diagnoses used to define events. **Table S3**. International Classification of Diseases [ICD] code 8/9/10 diagnoses and Classification of Surgical Procedures NOMESCO (Nordic Medico-Statistical Committee) codes used to define comorbidities. **Table S4**. Baseline characteristics of subjects with available BMI in sensitivity analysis. Categorical variables are presented as absolute and relative (percentages) frequencies, continuous variables as median and interquartile range.

## Data Availability

Anonymized personal data were obtained from national Swedish Registry holders after ethical approval and secrecy assessment. According to Swedish laws and regulations, personal sensitive data can only be made available for researchers who fulfil legal requirements for access to personal sensitive data. Contact Professor Niklas Hammar (niklas.hammar@ki.se) for questions about data access.
