# Supplementary material for: Impaired fasting glucose: a risk factor for atrial fibrillation and heart failure
Source: Cardiovasc Diabetol. 2021 Nov 24;20:227. doi: 10.1186/s12933-021-01422-3 (PMC8614025; doi:10.1186/s12933-021-01422-3)
Supplement: Supplementary file 1 — Additional file 1: Table S1. International Classification of Diseases [ICD] code 8/9/10 diagnoses and Classification of Surgical Procedures NOMESCO (Nordic Medico-Statistical Committee) codes used to define previous cardiovascular disease as exclusion criteria. Table S2. International Classification of Diseases [ICD] code 8/9/10 for diagnoses used to define events. Table S3. International Classification of Diseases [ICD] code 8/9/10 diagnoses and Classification of Surgical Procedures NOMESCO (Nordic Medico-Statistical Committee) codes used to define comorbidities. Table S4. Baseline characteristics of subjects with available BMI in sensitivity analysis. Categorical variables are presented as absolute and relative (percentages) frequencies, continuous variables as median and interquartile range. [file 12933_2021_1422_MOESM1_ESM.docx]

**Supplementary material**

**Supplementary table 1** - International Classification of Diseases [ICD] code 8/9/10 diagnoses and Classification of Surgical Procedures NOMESCO (Nordic Medico-Statistical Committee) codes used to define previous cardiovascular disease as exclusion criteria.

|  | **ICD-10** | **ICD-9** | **ICD-8** | **Surgical code** |
| --- | --- | --- | --- | --- |
| **Ischemic heart disease** | I20-I22 | 410-414 | 410-434 |  |
| **CABG/PCI** |  |  |  | 3066, 3067, 3080, 3127, FNA-FNE, FNG |
| **Heart failure** | I50 | 427-429 | 420-429 |  |
| **Atrial fibrillation** | I48 | 427.3 | 427.93, 427.94 |  |
| **Stroke** | I60-I69, G45 | 430-438 | 430-438 |  |
| **Haemorrhagic** | (I60-I62) |  |  |  |
| **Ischemic** | (I63-I64) |  |  |  |
| **Transitory**  **Ischemic attack** | (G45) |  |  |  |

Abbreviations: CABG=Coronary Artery Bypass Grafting; PCI=Percutaneous Coronary Intervention

**Supplementary table 2** - International Classification of Diseases [ICD] code 8/9/10 for diagnoses used to define events.

|  | **ICD-10** | **ICD-9** | **ICD-8** |
| --- | --- | --- | --- |
| Heart failure | I50 | 428.0 | 427.10  427.00 |
| Atrial fibrillation | I48 | 427.3 | 427.90  427.92  427.4 (cause of death) |

**Supplementary table 3** - International Classification of Diseases [ICD] code 8/9/10 diagnoses and Classification of Surgical Procedures NOMESCO (Nordic Medico-Statistical Committee) codes used to define comorbidities.

|  | **ICD-10** | **ICD-9** | **ICD-8** | **Surgical code** |
| --- | --- | --- | --- | --- |
| **Asthma/COPD** | J43-46 | 491, 492, 493, 496 | 490-493 |  |
| **Mitral stenosis** | I342, I050, I052, Q232 | 394, 424, 746.F | 394, 424 |  |
| **Mechanical prosthetic valve** |  | V43D |  | FGE00, FJF00, FKD00, FMD00 |
| **Liver disease** | K70-77 | 570 – 573 | 570 - 573 |  |
| **Chronic kidney disease** | N18 | 585 | 582, 792, Y29.08 |  |
| **History of cancer** | C-chapter | 140 – 165, 170-175, 179-208 | 140-163, 170-174, 180-207 |  |

Abbreviations: COPD=Chronic Obstructive Pulmonary Disease

|  | N | **Total**  N=55 390 | **Low**  n=1 622 (2.9%) | **Normal**  n=50 793 (91.7%) | **IFG**  n=1 537 (2.8%) | **Undiagnosed DM**  n=705 (1.3%) | **Diagnosed DM**  n=733 (1.3%) |
| --- | --- | --- | --- | --- | --- | --- | --- |
| Age | 55 390 | 45  (38-52) | 41  (35-48) | 44  (38-52) | 51  (44-58) | 54  (47-60) | 50  (42-56) |
| Female sex (%) | 55 390 | 28 075 (50.7) | 1 091 (67.3) | 26 014 (51.2) | 493 (32.1) | 184 (26.1) | 293 (40.0) |
| Blue collar worker | 54 529 | 32 061 (58.8) | 972 (61.0) | 29 278 (58.5) | 932 (61.4) | 427 (62.1) | 452 (62.8) |
| Born in Nordic countries | 55 390 | 52 041 (94.0) | 1 509 (93.0) | 47 761 (94.0) | 1442 (93.8) | 649 (92.1) | 680 (92.8) |
| Referred from occupational health care | 55 390 | 44 424 (85.7) | 1 268 (84.6) | 40 849 (85.9) | 1 217 (85.3) | 528 (81.5) | 562 (81.0) |
| BMI (kg/m2) | 55 390 | 24.0  (21.9-26.5) | 22.4  (20.6-24.6) | 24.0  (21.8-26.3) | 26.5  (24.2-29.5) | 28.0  (25.5-30.9) | 26.6  (23.4-29.8) |
| Kidney disease | 55 390 | 53 (0.1) | 0 | 51 (0.1) | 1 (0.1) | 0 | 1 (0.1) |
| Liver disease | 55 390 | 150 (0.3) | 7 (0.4) | 122 (0.2) | 6 (0.4) | 8 (1.1) | 7 (1.0) |
| Asthma/COPD | 55 390 | 340 (0.6) | 23 (1.4) | 292 (0.6) | 9 (0.6) | 7 (1.0) | 9 (1.2) |
| History of cancer | 55 390 | 915 (1.7) | 31 (1.9) | 828 (1.6) | 28 (1.8) | 18 (2.55) | 10 (1.4) |
| Mitral stenosis | 55 390 | 4 (0.0) | 0 | 3 (0.0) | 0 | 0 | 1 (0.) |
| Mechanical  valve-replacement | 55 390 | 1 (0.0) | 0 | 1 (0.0) | 0 | 0 | 0 |
| Fasting glucose, mmol/L | 55 390 | 4.8  (4.4 -5.2) | 3.7  (3.5-3.8) | 4.8  (4.5-5.1) | 6.3  (6.2-6.5) | 8.0  (7.3-9.9) | 8.0  (5.9-11.5) |
| Fructosamine, mmol/L | 48 481 | 2.1  (2.0-2.2) | 2.0  (1.9-2.2) | 2.1  (1.9-2.2) | 2.2  (2.0-2.3) | 2.5  (2.2-2.8) | 2.5  (2.2-2.9) |
| Total cholesterol, mmol/L | 55 390 | 5.6  (4.9-6.3) | 5.3  (4.6-5.9) | 5.6  (4.9-6.3) | 6.0  (5.2-6.8) | 6.1  (5.3-6.8) | 5.6  (4.9-6.5) |
| Triglyceride level, mmol/L | 55 390 | 1.0  (0.7-1.5) | 0.8  (0.6-1.2) | 1.0  (0.7-1.4) | 1.5  (1.0-2.3) | 1.9  (1.3-3.0) | 1.5  (0.9-2.3) |
| Apo-B/Apo-A1 ratio | 11 620 | 0.86  (0.69-1.07) | 0.76  (0.64-0.96) | 0.86  (0.68-1.06) | 0.96  (0.78-1.22) | 1.08  (0.89-1.32) | 0.96  (0.74-1.28) |
| Haemoglobin, g/L | 7 093 | 137.0  (130.0-146.0) | 132.0  (125.0-140.0) | 137.0  (129.0-146.0) | 144.0  (135.0-151.0) | 150.0  (140.5-159.5) | 141.0  (136.0-150.0) |
| WBC, 10^9/L | 6 593 | 6.1  (5.1-7.4) | 5.9  (4.8-7.9) | 6.0  (5.0-7.3) | 6.4  (5.4-7.8) | 6.8  (6.0-8.5) | 6.6  (5.6-7.8) |
| eGFR (ml/min/1.73 m^2^) <60 | 52 945 | 922 (1.7) | 26 (1.7) | 774 (1.6) | 65 (4.4) | 32 (4.8) | 25 (3.6) |
| Uric acid, umol/L | 51 695 | 278.0  (232.0-329.0) | 248.0  (211.0-301.0) | 277.0  (232.0-327.0) | 332.0  (285.0-387.0) | 322.0  (274.0-371.0) | 282.0  (230.0-339.0) |
| CRP, mg/L | 31 570 | 4.0  (1.0-6.0) | 3.0  (1.0-6.0) | 4.0  (1.0-6.0) | 4.0  (2.0-6.0) | 4.0  (1.0-7.0) | 4.0  (2.0-7.0) |
| Haptoglobin, g/L | 44 297 | 1.0  (0.9-1.2) | 1.0  (0.8-1.1) | 1.0  (0.9-1.2) | 1.1  (1.0-1.3) | 1.2  (1.0-1.4) | 1.1  (0.9-1.30) |

**Supplementary table 4** - Baseline characteristics of subjects with available BMI in sensitivity analysis. Categorical variables are presented as absolute and relative (percentages) frequencies, continuous variables as median and interquartile range.

Abbreviations: IFG=Impaired fasting glucose; DM=Diabetes mellitus; COPD=Chronic obstructive pulmonary disease; WBC=White blood cells.

**Laboratory measurements**

Information on fasting glucose, fructosamine, hemoglobin, LDL- HDL- and total cholesterol, triglycerides, apoB and apoA-1, uric acid, creatinine, C-reactive protein (CRP) and haptoglobin was used. Glucose was analysed from serum enzymatically with a glucose oxidase/peroxidase method based on a technique as per the GOD-PAP method using automated multichannel analysers. Levels of serum glucose were standardized to international standards of plasma glucose. Fructosamine levels were analysed using the Nitroblue Teterazolium (NBT) colorimetric technique using the same automatic multichannel analysers. Triglycerides and total cholesterol were analysed through enzyme techniques. Levels of apoB and apoA-1 was assessed through immunoturbidimetry. Creatinine levels were analysed with the non-kinetic alkaline picrate method (Jaffe´), from 1985 through 1992 by AutoChemistPRISMA and from 1993 through 1996 by DAX-96 analyser. Haemoglobin and white blood cells were measured by flow cytometry from whole blood with CoulterR STKS Haematology System (Coulter Corporation). CRP and haptoglobin was analysed with immunoturbi-dimetric assay.
